# Evaluation of the Role of p53 Tumour Suppressor Posttranslational Modifications and TTC5 Cofactor in Lung Cancer

**DOI:** 10.3390/ijms222413198

**Published:** 2021-12-07

**Authors:** Hasen Alhebshi, Kun Tian, Lipsita Patnaik, Rebecca Taylor, Pavel Bezecny, Callum Hall, Patricia Anthonia Johanna Muller, Nazila Safari, Delta Patricia Menendez Creamer, Constantinos Demonacos, Luciano Mutti, Mohamad Nidal Bittar, Marija Krstic-Demonacos

**Affiliations:** 1 School of Science, Engineering and Environment, University of Salford, Cockcroft Building 305, Manchester M5 4WT, UK; h.alhebshi@edu.salford.ac.uk (H.A.); n.safari@edu.salford.ac.uk (N.S.); d.p.menendezcreamer1@edu.salford.ac.uk (D.P.M.C.); 2 Institute of Biological Anthropology, School of Basical Medical Science, Jinzhou Medical University, Jinzhou 121001, China; kun.tian08@hotmail.com; 3 Blackpool Teaching Hospitals NHS Foundation Trust, Blackpool FY3 8NR, UK; dr.patnaik@bfwhospitals.nhs.uk (L.P.); rebecca.taylor73@nhs.net (R.T.); pavel.bezecny@bfwhospitals.nhs.uk (P.B.); professor.bittar@nhs.net (M.N.B.); 4 Cancer Research UK Manchester Institute, The University of Manchester, Alderley Park, Manchester SK10 4TG, UK; callum.hall@cruk.manchester.ac.uk (C.H.); patricia.muller@manchester.ac.uk (P.A.J.M.); 5 Division of Pharmacy and Optometry, Faculty of Biology, Medicine and Health, School of Health Sciences, The University of Manchester, Stopford Building, 3.124 Oxford Road, Manchester M13 9PT, UK; constantinos.demonacos@manchester.ac.uk; 6 Center for Biotechnology, Sbarro Institute for Cancer Research and Molecular Medicine, College of Science and Technology, Temple University, Philadelphia, PA 19122, USA; luciano.mutti@temple.edu

**Keywords:** p53, TTC5, lung cancer

## Abstract

Mutations in the p53 tumor suppressor are found in over 50% of cancers. p53 function is controlled through posttranslational modifications and cofactor interactions. In this study, we investigated the posttranslationally modified p53, including p53 acetylated at lysine 382 (K382), p53 phosphorylated at serine 46 (S46), and the p53 cofactor TTC5/STRAP (Tetratricopeptide repeat domain 5/ Stress-responsive activator of p300-TTC5) proteins in lung cancer. Immunohistochemical (IHC) analysis of lung cancer tissues from 250 patients was carried out and the results were correlated with clinicopathological features. Significant associations between total or modified p53 with a higher grade of the tumour and shorter overall survival (OS) probability were detected, suggesting that mutant and/or modified p53 acts as an oncoprotein in these patients. Acetylated at K382 p53 was predominantly nuclear in some samples and cytoplasmic in others. The localization of the K382 acetylated p53 was significantly associated with the gender and grade of the disease. The TTC5 protein levels were significantly associated with the grade, tumor size, and node involvement in a complex manner. SIRT1 expression was evaluated in 50 lung cancer patients and significant positive correlation was found with p53 S46 intensity, whereas negative TTC5 staining was associated with SIRT1 expression. Furthermore, p53 protein levels showed positive association with poor OS, whereas TTC5 protein levels showed positive association with better OS outcome. Overall, our results indicate that an analysis of p53 modified versions together with TTC5 expression, upon testing on a larger sample size of patients, could serve as useful prognostic factors or drug targets for lung cancer treatment.

## 1. Introduction

Lung cancer is a common type of cancer as well as a frequent cause of death in both men and women worldwide. In the UK in 2017, lung cancer was the third most common cancer (Cancer Research, UK. Available online: https://about-cancer.cancerresearchuk.org/about-cancer/lung-cancer/about, accessed on 7 December 2021). Each year, more than a million new cases of lung cancer are diagnosed worldwide [1]. Causes of lung cancer include smoking, air pollution, asbestos, and genetic elements [2]. Lung cancer can be categorized into two types depending on its histological appearance. These types are non-small cell lung cancer (NSCLC) and small cell lung cancer (SCLC). SCLC makes up about 20–25% of lung cancers [3]. Moreover, the grading system (grades 1–3 on the basis of histology of the tumor) and the TNM staging (T-tumor size, N-node involvement, and M-distant metastases) are standardly used in the clinic to inform prognosis and therapy. Most patients are diagnosed at the stage of advanced disease and despite continued improvement in therapy, the five-year survival rate remains low [4,5].

The p53 tumor suppressor gene is frequently found mutated in lung cancer [6,7,8]. Mutations of p53 lead to either inactivation or creation of p53 dominant-negative forms that often display oncogenic potential [9,10]. p53 is a transcription factor that mediates cellular response to stress controlling cell proliferation, promoting cellular response to DNA damage, DNA repair, and eliminating cells damaged beyond repair through apoptosis and other types of cell death [7,11]. If p53 or pathways involved in its regulation are altered by mutations, this results in uncontrolled cell division, increased mutations, and cancer. Given that p53 protein is pivotal for cancer progression, its function has been extensively researched [12]. Numerous p53 mutations and its loss have been identified in lung cancer and suggested to contribute to poor survival rate [13,14,15,16].

p53 activity is regulated at several levels including protein stability and transcriptional control. P53 posttranslational modifications play an important role in this control and include phosphorylation, acetylation, ubiquitination, and several more. Phosphorylation of p53 plays a major role in its protein stability and transcriptional activation, notably controlling interaction with its negative regulator MDM2 that promotes p53 degradation [17,18,19]. In particular, serine 46 phosphorylation directs the p53 protein to promoters of genes that regulate apoptosis [20]. Transcriptional coactivators and histone acetyltransferases p300/CREB-binding protein (CBP) as well as MOZ were suggested to acetylate p53 at K382 [21] and members of the sirtuin family including SIRT-1 were implicated in deacetylation [22]. The lysine acetylation at these sites is linked to p53 protein stability, transcriptional activation, and the p53 mediated control of cell cycle arrest and apoptosis. Furthermore, p53 can be modified in many ways by various proteins at different locations within p53 in an independent manner, which contributes to the fine tuning of p53 protein activity [23].

Another level of p53 activity control includes transcriptional cofactors. The histone acetyltransferase (HAT) p300 is a p53 coactivator that acts by acetylating p53 and thereby increasing p53 activity. TTC5/STRAP is a p300 interacting protein that is found in a complex with JMY and p300 and leads to p53 coactivation through p53 stabilization and increase in p53 mediated transcriptional activation [24,25,26]. TTC5 is composed of six tandem tetratricopeptide repeat (TPR) motifs, which are 34 amino acid long motifs degenerate in nature, mediating protein–protein interaction and are found in a variety of proteins [27]. TTC5/Strap is a stress responsive co-chaperone involved in the modulation of p53, HSF1, GR, and AP1 transcriptional activity as well as p53 and GR protein stability [28,29,30]. TTC5 interacts with mitochondrial ATP synthase to downregulate ATP production, and it has been reported to increase the apoptotic effects of mitochondrial p53 [31]. TTC5 augments p300 mediated acetylation [26]. TTC5 is involved in the DNA damage response and is phosphorylated by ATM on S203, which increases its nuclear accumulation, whereas Chk2 phosphorylation on S221 augments protein stability [26,32]. JMY and TTC5 are involved in cell adhesion and control the tubulin mRNA stability, thereby inhibiting actin nucleation and regulation of autophagy [33,34,35,36,37]. Interestingly, TTC5 prevents apoptosis of acute myeloid leukaemia cells [38].

In this report, the potential of total and modified p53, TTC5, and SIRT1 as biomarkers in lung cancer patients was analysed. Significant associations between clinicopathological features and expression of the studied proteins were identified and an unusual cytoplasmic location of acetylated p53 was observed. Results suggest that a combined analysis of p53 modification and cofactors such as TTC5 associations with clinicopathological features may provide new potential prognostic factors and drug targets in lung cancer.

## 2. Results

### 2.1. Association between Protein Expression and Clinicopathological Features in Lung Cancer Patients

**Figure 1:**
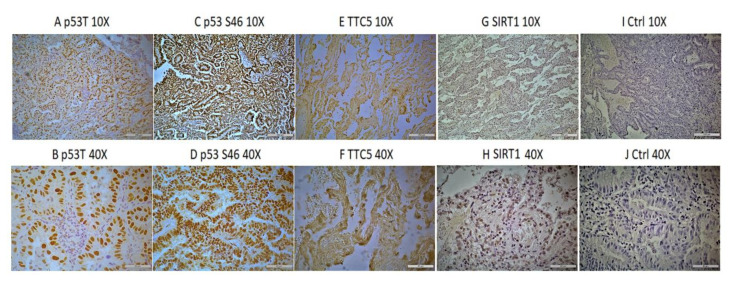
Example of the studied proteins staining in human lung cancer tissues. (**A**,**B**) positive staining of total p53 protein (p53T); (**C**,**D**) p53 phosphorylated at serine 46 (p53 S46); (**E**,**F**) TTC5 protein; and (**I**,**J**) SIRT1 at magnification 10× (**A**,**C**,**E**,**G**,**I**) and 40× (**B**,**D**,**F**,**H**,**J**). (**I**,**J**) is a control staining of lung cancer tissue with IgG antibody used as primary antibody. Scale bars indicate 250 µm for 10× and 62 µm for 40× magnification.

To determine the p53 expression in lung cancer patients, 250 cases of tissue microarrays (TMA) and human lung cancer tissue section slides were analysed by using the immunohistochemistry (IHC) technique. We chose to study total p53 and p53 phosphorylated at S46 that is the main phosphorylation site important for p53-mediated control of proapoptotic genes [20]. In addition, K382 is acetylated by p300 histone acetyltransferase [39]. TTC5 serves as a cofactor of p53, it interacts with p300 and controls acetylation of its targets [25,26,27]. SIRT1 is a deacetylase involved in p53 posttranslational modifications, and its status was followed on a subset of slides. An example of protein staining is illustrated in Figure 1, where staining results demonstrate the positive staining of the p53 total and phosphorylated at serine 46 protein respectively (Figure 1A–D), TTC5 (Figure 1E,F), SIRT1 (Figure 1G,H) as well as the negative control where IgG antibody was used (Figure 1I,J).

The relationship between p53 expression in the lung cancer patients’ tissues and clinicopathological features was investigated (Table 1). Normal lung tissue sections were negative for staining specific for p53 protein expression. Out of the 250 lung cancer cases, 163 positively stained for p53 in the nuclei of the tumor cells. No significant association was observed between the p53 expression and age, gender or stage (Supplementary Table S1). Total p53 protein expression intensity did not show association with expression intensity of K382 and S46 p53 modified versions, TTC5, or SIRT1 expression (Supplementary Table S1). Positive p53 staining was significantly associated with cancer grade (70.6% of grade 3 were p53 positive, compared to 65.9% of grade 2, and 33.3% of grade 1, *p* = 0.001). Therefore, p53 presence detected with the p53 specific antibody is associated with the higher grade of lung cancer (Table 1).

The incidence of positive staining for p53 acetylated at lysine 382 expression was 171 (68.4%), out of which 139 (73.2%) samples were positive in male and 32 (53.3%) in female patients (*p*-value 0.004), suggesting male patients are more likely to have tumours that stain positively for K382. In addition, a significant association was also found between K382 expression and cancer grade (*p*-value 0.039), as well as between K382 and total p53 expression intensity (0.044) (Table 2).

It was observed that K382 localization is different in different samples; some had predominant nuclear (Supplementary Figure S1A,B), and some had predominant cytoplasmic staining (Supplementary Figure S1C,D). This was associated significantly with gender and grade of cancer (*p*-value 0.007 and 0.024 respectively) (Table 3). Nuclear staining of K382 was mostly associated with tumours in male patients. Grade 2–3 tumours had the highest K382 nuclear staining, whereas grade 1–2 had the highest cytoplasmic K382 staining. No statistically significant associations were seen when cytoplasmic and nuclear staining of K382 acetylated p53 was analysed together with S46, TTC5, and SIRT1 protein expression (Supplementary Table S2). In summary, p53 protein acetylated at K382 was more frequently found in male patients and was observed in both cytoplasm and the nucleus.

The p53 phosphorylated at Ser-46 protein expression and their potential prognostic significance was investigated next (Table 4). The positive expression of phosphorylated p53 at Ser46 was detected in 219 out of 250 cases. A significant association was indicated between the p53 phosphorylated at Ser46 and the pathological grade of cancer; however, there was no clear divide in expression of this protein between high and low cancer grade (*p*-value 0.019). No significant association was observed between other parameters studied and expression of phosphorylated p53 at Ser46 (Supplementary Table S3).

The incidence of TTC5 expression indicated that 183 cases positively stained for TTC5, mostly found in the cytoplasm of the cancer cells. Significant associations were observed between the TTC5 protein and cancer grade (*p* = 0.039), although it is difficult to conclude what type of association this represents, given different trends for single and double grades (Table 5, Supplementary Table S4). Significant associations were found for TTC5 protein expression and TNM stage (*p* < 0.001). Post-hoc comparisons of pairs within each of these factors showed that tumours at stage M0 had significantly higher TTC5 expression than those at stage MX (87.5% positive expression compared to 25.0%, adjusted *p* = 0.004). However, tumours at stage T2 had significantly higher TTC5 expression than those at T1 (88.0% vs 54.8%, adjusted *p* = 0.0116). Other pairwise comparisons failed to achieve significance, though there is evidence that a larger sample may also yield increasingly high TTC5 expression for higher T-stages. There was a significant association with N status and TTC5 protein expression (*p* value 0.013), and the pairwise comparisons using the Fisher exact test by R platform suggested that difference was significant when N0 was compared with N1. It seems that TTC5 is positively associated with higher tumour stage (T2 versus T1) and nodes involvement (N1 versus N0), whereas association with distant metastasis (M status) is inconclusive due to lack of a representative sample, highlighting the need for a larger sample analysis.

SIRT1 expression was evaluated in 48 lung cancer patients, and significant correlations were found with S46 intensity (*p*-value 0.004); higher S46 intensity was associated with higher SIRT1 expression, whereas SIRT1 protein expression was inversely associated with positive TTC5 staining (*p*-value 0.019 (Table 6). These results suggest a potentially important link between p53 phosphorylation, TTC5, and SIRT1 function in lung cancer.

### 2.2. Kaplan-Meier Plots

**Figure 2:**
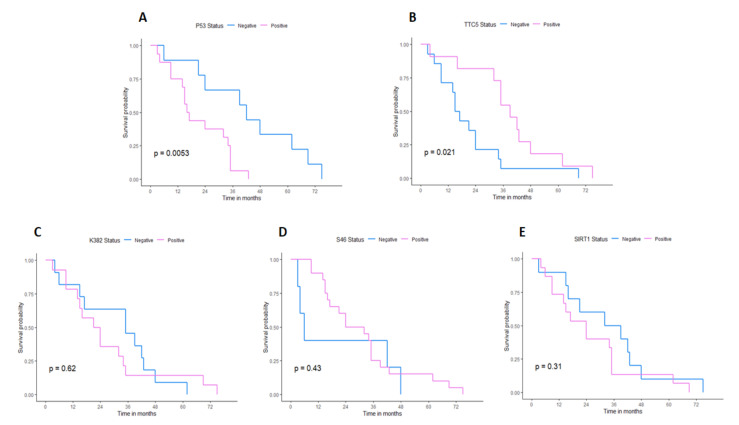
Kaplan-Meier survival analysis. The subgroup KM curves are represented by different colours. The *p*-values are shown in the figure. The X axis shows the overall survival (OS) time in month. KM curves of the subgroups for total p53 (**A**), TTC5 (**B**), p53 K382 (**C**), p53 S46 (**D**), and SIRT1 (**E**) are shown.

Kaplan-Meier (KM) estimates were calculated and plotted to determine whether the presence of staining (Figure 2), the degree of staining, or cytoplasmic/nuclear K382 staining (Supplementary Figure S2) were associated with patient survival. While K382, S46, and SIRT1 show no evidence of association with survival, samples stained with antibodies against TTC5 had significantly better survival rate than those negative for TTC5 (log-rank test *p* = 0.021), and positive p53 staining was significantly associated with worse survival than those negative for p53 (log-rank test *p* = 0.005). Given that WT p53 is expressed at very low levels in cancers, it is generally appreciated that high expression of p53 in tumours signifies expression of a mutant form of p53 that may have dominant negative and oncogenic characteristics through the gain of new function and other processes [40,41]. These results suggest that positive TTC5 staining was associated with longer survival, whereas p53 positive staining was associated with worse survival.

### 2.3. Cox Analysis

To determine which proteins expression levels (negative, moderate, or high), subcellular location (nuclear, cytoplasmic, or both), or expression status (positive or negative) are associated with the hazard of death, Cox proportional hazards regression univariate analysis was used (Table 7). Results are shown as a hazard ratio (HR) and a 95% confidence interval for the HR; HR < 1 indicates the factor is associated with reduced risk of death, a better prognosis. Conversely, an HR > 1 indicates an increased hazard of death or a worse prognosis.

The highest negative and significant association with survival was observed between TTC5 status (HR 0.372, *p*-value 0.0221) and TTC5 expression (HR 0.53, *p*-value 0.0249), suggesting that TTC5 could be a good prognostic factor. The highest positive association that was statistically significant was with the expression and status of p53 protein (HR 1.95, *p*-value 0.0041; HR 4.29, *p*-value 0.0035), suggesting that p53 was a negative prognostic factor. Expression, status, and localization of other proteins didn’t reach statistical significance as indicated by *p*-values higher than 0.05.

Given that p53 and TTC5 detection was limited to protein expression, association analysis of TTC5 gene expression and p53 gene expression or mutation status in the TCGA lung cohort was carried out (Supplementary Figure S3). Analysis indicated there was no association between p53 gene expression or TTC5 gene expression, and TTC5 gene expression was not associated with mutant p53 or WT status.

### 2.4. Immunofluorescence Results

Since IHC results indicated that the acetylated form of p53 at lysine 382 in some tissue samples from lung cancer patients was detected in the cytoplasm, the expression of this isoform was analysed in cancer cell lines using the immunofluorescence technique. The H2170 and A549 lung cancer cell line, osteosarcoma cell lines (U2OS), as well as BEAS 2B noncancerous lung cell line were treated for 24 h with 20 µM Etoposide, the cells were fixed and nuclei stained by DAPI (blue), P53 specific DO-1, anti-K382, and anti-TTC5 antibodies (Supplementary Figures S4–S7).

Most cell lines showed predominantly nuclear staining when the anti-K382 antibody was used; in some cells, residual cytoplasmic staining was observed with K382 antibody in the BEAS 2B cell line, although, the significance of this observation is not clear. In A549 and U2OS cells, the TTC5 protein was observed in both the nucleus and cytoplasm (Supplementary Figure S6). These results suggest that the acetylated p53 was expressed generally in the nucleus, whereas the TTC5 protein was expressed in the nucleus and the cytoplasm in cell lines investigated.

## 3. Discussion

In this report, we demonstrate that p53, its modified versions, and the TTC5 cofactor are associated with several clinical parameters, suggesting that the p53 acetylation pathway has an important role in lung cancer development. Significant association was detected between the p53 total, S46, K382, and TTC5 protein expression levels and the grade of lung cancer. The SIRT1 protein expression was inversely associated with TTC5 expression and positively associated with p53 phosphorylated at S46. KM plots indicated that p53 was associated with worse survival, whereas TTC5 was associated with a better survival outcome.

Lung cancer is an important contributor to cancer related deaths in part due to a lack of biomarkers to detect early stages of this disease. In breast cancer, mutant p53 expression is detected in the early stages and signifies worse progression [42]. In lung cancer, p53 tumour suppressor is often mutated and is an important regulator of response to DNA damage or elimination of damaged cells through the apoptotic process. Due to its pivotal role, it was extensively studied in lung cancer using the immunohistochemical method to detect p53 protein that was usually detected in the nucleus and suggestive of presence of mutations. There are over 60 studies investigating the prognostic value of p53 detection in NSCLC [43]. However, the potential of p53 acetylation and TTC5 co-factors as new potential prognostic factors to improve patients’ stratification into high and low risk groups remains to be investigated.

Our results suggest that p53 total protein as well as its modified versions correlate with the grade of cancer, and total p53 positivity was associated with a markedly reduced patient survival. This is in line with other published reports indicating that mutated or overexpressed p53 detection was an indicator of poor prognosis for patients with adenocarcinoma [43]. Unexpectedly, our report suggests that in some patients K382 acetylated p53 isoform was detected in the cytoplasm (Supplementary Figure S1). This is surprising given the nuclear location of p53 protein in most reports. Our results suggest that studied cell lines show predominantly nuclear staining; in some cells, and under certain experimental conditions, residual cytoplasmic staining was observed when the K382 antibody was used in in the BEAS 2B cell line. Although the significance of this observation is not clear, acetylated p53 is known to have cytoplasmic functions, and its accumulation in the cytoplasm has been previously reported [21,44,45,46].

Many of the studies have examined p53 expression; however, the acetylation of mutant p53 has not been thoroughly investigated [47]. This will be important to know as the vast majority of the tumours with high levels of p53 are actually expressing a mutant p53. Perhaps mutant p53 is more acetylated in the cytoplasm than the nucleus. Future experiments addressing mutant p53 status by sequencing and analysis of p53 posttranslational modifications should answer these questions. In addition, MDM2 and ARF14 have been shown to prevent cytoplasmic localization of acetylated p53, possibly suggesting that differential levels of MDM2 and ARF14 in these samples could determine cytoplasmic expression [48]. It will be interesting to determine levels of MDM2 and ARF14 in parallel in future studies and to study the effect of acetylation on mutant p53.

Another unexpected finding was that more cases were positive for K382 p53 staining than for total p53 staining in males (139 vs. 127 cases, Table 1). This discrepancy might be due to antibody specificity that potentially can recognize other antigens and not only p53. It is also possible the abovementioned observation is due to the difference in the DO-7 and K382 p53 specific antibodies affinities for the antigen. However, it could also point to the presence of N-terminal isoforms of p53. For total p53 levels, the well-established DO-7 antibody was used, which is a monoclonal antibody that recognizes a domain in the extreme N-terminus of p53, while K382 acetylation happens on the p53 C-terminus. N-terminal truncations of p53 (Δ40, or Δ133) have been reported to play a role in cancers [49], but it is unknown whether they play a role in lung cancer specifically. Our results warrant further investigation into N-terminal truncated variants and acetylation in this cohort of patients.

An additional interesting finding in our manuscript is that acetylated p53 was proportionally higher in male patients than in female patients (Table 1). Recently Haupt et al. demonstrated that there is sex-disparity in p53 mutations in cancers [50]. In this work, it is suggested that negative p53 regulator genes expressed on the X-chromosome are involved in reduced survival of males from cancers. Perhaps some of these regulators are involved in p53 acetylation and could explain the findings in this manuscript.

One factor that connects p53 phosphorylation and acetylation is the p300 histone acetyltransferase. P53 phosphorylation status is important for p300 recruitment to p53 and p53 acetylation. This is through a multiprotein complex involving several other factors including JMY and TTC5 [26]. TTC5 has been suggested to facilitate p300 mediated acetylation of p53. The results here show a significant and complex association of TTC5 protein expression with the grade, stage of cancer, and OS. TTC5 seems to be positively associated with tumor size and node involvement, whereas a potential association with metastasis is difficult to interpret due a difference in number of samples analysed in different categories. Furthermore, TTC5 was associated with a better overall survival. It is possible that this is due to different methods/histological analyses used in the survival and grading analysis indicating a need for larger sample investigations in future research. Several reports suggest that TTC5 controls elements of cytoskeleton actin and tubulin [51], which are crucial components of migratory and metastatic potential of cancer cells potentially explaining association with metastatic and node status. TTC5 expression was also negatively associated with the intensity of SIRT1 protein expression. This inverse association with SIRT1 is in line with the role of TTC5 in facilitating p53 acetylation.

Overall, our results suggest that analysis of p53 posttranslational modifications, in combination with TTC5 expression, upon further testing on a larger number of patients, may provide further insight into the role of p53 pathway in lung cancer biology. These observations can be exploited to simultaneously target multiple pathways associated with p53 [52,53], including TTC5-mediated control of cytoskeleton, to overcome chemotherapy resistance to tubulin binding agents [51,54,55]. In addition, given the potential association of TTC5 with better overall survival, upon confirmation of this finding on larger number of patients, this observation can be used to stratify patients into high or low risk group and inform future clinical decisions. Finally, TTC5 may have a role in the process of antigen presentation, and together with p53 posttranslational modifications, may be essential in the selection of patients for immunotherapy. Taken together, these observations will facilitate future clinical applications in the treatment of patients with lung cancer.

## 4. Materials and Methods

### 4.1. Patients and Samples

In total, 202 lung cancer patients’ TMA tissue samples were purchased from US Biomax, Inc., Derwood, MD, USA as formalin-fixed paraffin-embedded tissue sections. Additionally, another 48 lung cancer tissue sections were included in this study and obtained from Blackpool Teaching Hospitals NHS Foundation Trust (BTHNFT). Most of the 250 lung cancer tissue sections were adenocarcinomas and squamous cell carcinoma (93 adenocarcinoma (37.2%), 132 squamous cell carcinoma (52.8%), 1 carcinoid (0.4%), 3 atypical carcinoid (1.2%), 8 small cell carcinoma (3.2%), 3 large cell carcinoma (1.2%), 4 bronchioloalveolar carcinoma (1.6%), 3 mucinous adenocarcinoma (1.2%), and 3 adenosquamous carcinoma (1.2%)) (Supplementary Table S5). In total, every tissue section was 4–5 µm thick with a 1.5mm diameter for the TMA cores on the slides. The tissue specimens in this study were used to identify the associations between the differentially expressed target proteins (total p53, p53 acetylated at K382, p53 phosphorylated at S46, SIRT1, and TTC5 proteins) and clinicopathological data. Clinical information was obtained by reviewing medical records, which included the patient’s age, gender, cancer histological grade, the TNM (primary tumor, lymph nodes, and metastasis) staging and stage groups I–IV. The 48 samples from BTHNFT had survival data. Ethical approval was granted by the University of Salford ethics committee and from the National Research Ethics Committee (NREC, LAMMA study).

### 4.2. Cell Lines

Human lung adenocarcinoma A549, an immortalized human lung epithelial cell line BEAS-2B, human squamous cell carcinoma H2170, and U2OS human osteosarcoma cancer cell lines were purchased from American Type Culture Collection (ATCC; Manassas, VA, USA); Mero-14 human mesothelioma cell line was a gift from Prof. Landi (University of Pisa). Cells were maintained at 5% CO_2_ and 37°C in RPMI 1640 medium (Sigma Aldrich, Gillingham, UK), which contained 10% fetal calf serum (Gibco, Paisley. UK) and 1% penicillin/streptomycin 10,000 U/mL (Sigma Aldrich, Gillingham, UK). Cells were treated with 20 µM topoisomerase II inhibitor etoposide [39] (Sigma Aldrich, Gillingham, UK) for 24 h.

### 4.3. Immunohistochemistry Approach (IHC)

Immunohistochemical staining was performed using a two-step indirect immunohistochemistry protocol. Histo-clear1 and 2 solutions were used to remove the paraffin wax followed by rehydration. For antigen retrieval, slides were placed in the microwave for 15–20 min at 310–440 W and then covered with the Tris-EDTA Buffer pH 9.0 (10 mM Tris Base, 1 mM EDTA, 0.05% Tween-20) or Tri-sodium citrate buffer PH 6.0 (10 mM Sodium Citrate, 0.05% Tween-20, pH 6.0), depending on the antibody used in the experiment. The blocking of endogenous enzymes was performed by incubating the slides with the 0.3% of hydrogen peroxide (H_2_O_2_) for 15 min. Samples that underwent the same procedure but with the addition of the IgG antibody were considered negative control.

The antibody for p53 (DO-7, 1:100 dilution) was purchased from DAKO (CA, USA), Phosphorylated p53 (Ser46 (1:100 dilution) was obtained from Abnova (Taipei, Taiwan), acetylated p53 (K382) (1:50 dilution) was from Abcam, Cambridge, UK. Antibodies for TTC5 (1:100 dilution) and SIRT1 (1:100 dilution) were ordered from Abcam (Cambridge, UK). The immunohistochemical staining patterns were reviewed by two researchers including a pathologist and calculated as staining intensities (0–3).

### 4.4. Immunofluorescence

The A549 cells were cultured on the slides as described previously [26] and treated with 20 µM Etoposide for 24hrs. Cells were first washed 3 times with cold PBS, then fixed with 4% formaldehyde for 15 min, followed by 5 min incubation in 1% Triton/PBS, and then washed 3 times with PBS. Then, 1% BSA (Bovine Serum Albumin) was used for blocking procedure, for one hour. TTC5 rabbit polyclonal antibody and mouse monoclonal antibody against p53 acetylated at K382 were used. The TTC5 antibody (1:200 dilution) was mixed for one hour with K382 antibody (1:1000 dilution) in the 1% BSA. The slides were washed with 1% BSA, then incubated for an hour with secondary antibodies (green fluorescence for the anti-TTC5 antibody and red fluorescence for the anti-K382 antibody (dilution 1:1000)). DAPI staining was used to stain nuclei and slides were analysed using the Leica microscope.

### 4.5. TTC5 and p53 Coexpression Analysis

Data were obtained from the UCSC Xena browser (https://xenabrowser.net/, accessed on the 7 December 2021). Expression data for TTC5 and TP53 were downloaded from the TCGA LUNG database. Using R, the expression levels of TP53 and TTC5 mRNA were plotted on a scatter graph using scatter.smooth. Using the same dataset, TTC5 expression was subdivided by samples with mutant or wild-type TP53. The expression data of TTC5 for each subgroup were then displayed as a boxplot using boxplot.

### 4.6. Statistical Analysis

SPSS version 25 and R version 3.6.2. were used for statistical analysis. The following R packages were used in R script for the survival analysis and further PH hypothesis verification: “survival”, “surviminer”, and “rcompanion”. Chi-square tests and Fisher’s exact tests were utilized to determine the association of expression of proteins with clinical parameters such as age, gender, tumor grade and disease stage, primary tumor size, and metastases to other organs and lymph nodes. Post-hoc pairwise tests of TTC5 expression at different stages were adjusted for multiple comparisons using Bonferroni corrected *p*-values. Statistical significance was determined based on a significance level of *p*-value ≤ 0.05.

### 4.7. Survival Analysis

All survival analyses were performed using the survival and survminer packages on the R platform [56,57]. Kaplan-Meier estimates of survival probability for sub-groups of parameters including p53, K382, S46, SIRT1, and TTC5 expression level, status, and K382 localization were calculated, and sub-group differences tested using a log-rank test. Kaplan-Meier curves were also plotted for visualization of these differences.

Univariate Cox Proportional Hazards models were fitted to each of the status/expression parameters under investigation to estimate the marginal strength of each association with survival, and those found to be significantly associated with survival (with *p*-value < 0.05) were further included in a multivariate Cox PH model. The assumption of proportional hazards was satisfied; hazard ratios for each parameter are presented along with their 95% confidence interval.

## Figures and Tables

**Table 1:** Association of total p53 (Tp53) with clinicopathological features.

	TP53		
Characteristic	N°	Positive	*p*-Value
Cancer Grade			0.001
Grade 1	6 (3.4%)	2 (33.3%)	
Grade 2	88 (50%)	58 (65.9%)	
Grade 3	51 (29.0%)	36 (70.6%)	
Grade 1-2	20 (11.4%)	5 (25.0%)	
Grade 2-3	11 (6.3%)	10 (90.9%)	

**Table 2:** Association of acetylated p53 (p53) form (K382) with clinicopathological features.

	K382		
Characteristic	N°	Positive	*p*-Value
Gender			0.004
Male	190 (76.0%)	139 (73.2%)	
Female	60 (24.0%)	32 (53.3%)	
Cancer Grade			0.039
Grade 1	6 (3.4%)	4 (66.7%)	
Grade 2	88 (50%)	67(76.1%)	
Grade 3	51 (29.0%)	31 (60.8%)	
Grade 1-2	20 (11.4%)	17 (85%)	
Grade 2-3	11 (6.3%)	11 (100%)	
P53 Intensity			0.044
Negative	87 (34.8%)	52 (59.8%)	
Weak	74 (29.6%)	50 (67.6%)	
Moderate	41 (16.4%)	29 (70.7%)	
Strong	48 (19.2%)	40 (83.3%)	

**Table 3:** Association of the acetylated p53 (K382) subcellular localization with clinicopathological features.

	K382		
Characteristic	N°	Positive	*p*-Value
Gender			0.004
Male	190 (76.0%)	139 (73.2%)	
Female	60 (24.0%)	32 (53.3%)	
Cancer Grade			0.039
Grade 1	6 (3.4%)	4 (66.7%)	
Grade 2	88 (50%)	67(76.1%)	
Grade 3	51 (29.0%)	31 (60.8%)	
Grade 1–2	20 (11.4%)	17 (85%)	
Grade 2–3	11 (6.3%)	11 (100%)	
P53 Intensity			0.044
Negative	87 (34.8%)	52 (59.8%)	
Weak	74 (29.6%)	50 (67.6%)	
Moderate	41 (16.4%)	29 (70.7%)	
Strong	48 (19.2%)	40 (83.3%)	

**Table 4:** Association of the phosphorylated p53 (Ser-46) with clinicopathological features.

	S46		
Characteristic	N°	Positive	*p*-Value
Cancer grade			0.019
Grade 1	6 (3.4%)	6 (100%)	
Grade 2	88 (50.0%)	84 (95.5%)	
Grade 3	51 (29.0%)	42 (82.4%)	
Grade 1-2	20 (11.4%)	20 (100%)	
Grade 2-3	11 (6.3%)	11 (100%)	

**Table 5:** Association of TTC5 protein levels with clinicopathological features.

	TTC5		
Characteristic	N°	Positive	*p*-Value
Cancer grade			0.039
Grade 1	6 (3.4%)	5 (83.3%)	
Grade 2	88 (50.0%)	73 (83.0%)	
Grade 3	51 (29.0%)	43 (84.3%)	
Grade 1-2	20 (11.4%)	12 (60.0%)	
Grade 2-3	11 (6.3%)	6 (54.5%)	
TNM Stage			
T1	31 (20.3%)	17 (54.8%)	<0.001
T2	92 (60.1%)	81 (88.0%)	
T3	23 (15.0%)	21 (91.3%)	
T4	7 (4.6%)	5 (71.4%)	
M0	132 (93.0%)	115 (87.1%)	<0.001
M1	2 (1.4%)	1 (50.0%)	
MX	8 (5.6%)	2 (25.0%)	
N0	88 (59.5%)	64 (72.7%)	0.013
N1	55 (37.2%)	52 (94.5%)	
N2	4 (2.7%)	3 (75%)	
NX	1 (0.7%)	1 (100%)	
SIRT1 Intensity			0.044
Negative	15 (31.3%)	11 (73.3%)	
Weak	3 (6.3%)	1 (33.3%)	
Moderate	9 (18.8%)	2 (22.2%)	
Strong	21 (43.8%)	7 (33.3%)	

**Table 6:** Association of SIRT1 protein levels with clinicopathological features.

	SIRT1		
Characteristic	N°	Positive	*p*-Value
S46 Intensity			0.004
Negative	8 (16.7%)	4 (50.0%)	
Weak	11 (22.9%)	4 (63.4%)	
Moderate	15 (31.3%)	11 (73.3%)	
Strong	14 (29.2%)	14 (100%)	
TTC5 Intensity			0.019
Negative	27 (56.3%)	23 (85.2%)	
Weak	16 (33.3%)	8 (50.0%)	
Moderate	5 (10.4%)	2 (40.0%)	
Strong	/	/	

**Table 7:** Univariate COX analysis. Status, expression (exp), and subcellular localization (loc) are shown.

	HR (95% CI for HR)	*p*-Value
Status TTC5	0.372 (0.158–0.874)	0.0221
Exp. TTC5	0.53 (0.297–0.947)	0.0249
Status S46	0.693 (0.251–1.91)	0.493
Exp. S46	0.963 (0.619–1.5)	0.869
Status K382	1.24 (0.537–2.88)	0.611
Loc. K382	1.26 (0.771–2.05)	0.365
Exp. K382	1.34 (0.664–2.69)	0.423
Status SIRT1	1.59 (0.685–3.68)	0.275
Exp. SIRT1	1.37 (0.967–1.93)	0.0748
Status p53	4.29 (1.48–12.4)	0.0035
Exp. p53	1.95 (1.24–3.06)	0.0041

## Supplementary Materials

The following are available online at https://www.mdpi.com/article/10.3390/ijms222413198/s1.
