# Supplementary material for: Evaluation of the Role of p53 Tumour Suppressor Posttranslational Modifications and TTC5 Cofactor in Lung Cancer
Source: Int J Mol Sci. 2021 Dec 7;22(24):13198. doi: 10.3390/ijms222413198 (PMC8707832; doi:10.3390/ijms222413198)
Supplement: Supplementary file 1 [file ijms-22-13198-s001.zip › Supplementary material revised Alhebshi et al 07122021.pdf]

Supplementary figures Alhebshi et al

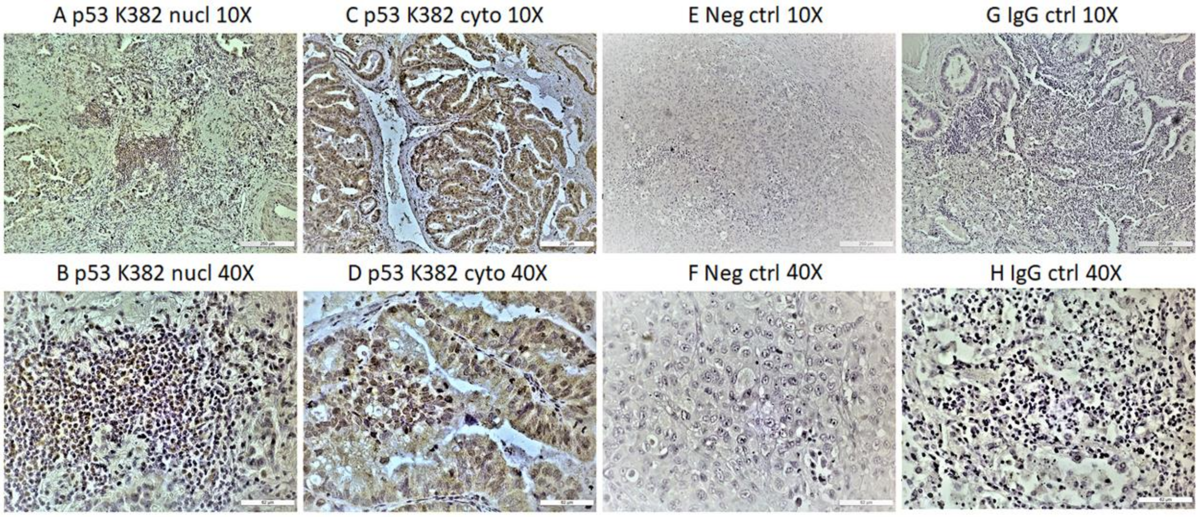

Supplementary figure S1 Immunostaining of the acetylated p53 at K382 protein in paraffin-embedded human lung cancer tissues using anti-acetyl-p53 (K382) antibody. A, B shows nuclear and cytoplasmic positive staining (brown staining). C, D strong cytoplasmic staining only, showing the location and the intensity of this protein. E, F negative staining obtained when lung cancer tissue was stained with antibody against acetylated p53 at K382. G, H negative control sample obtained when lung cancer tissue was stained with IgG primary antibody. Scale bars indicate 250µm for 10X and 62µm for 40X magnification.

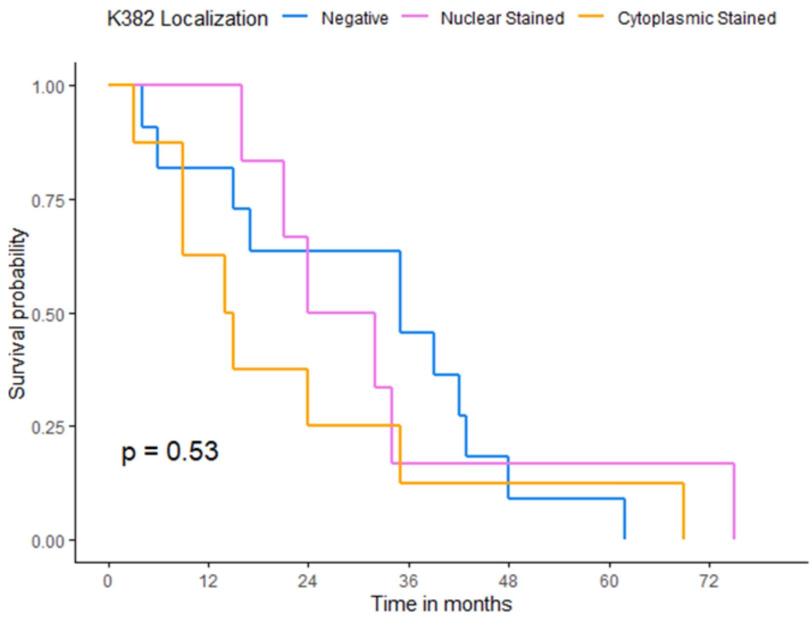

Supplementary figure S2 Kaplan-Meier plot – subcellular location of p53 acetylated on K382. The subgroup KM curves are represented by different colour. The p-value is shown in the figure. The X axis shows the overall survival time in months.

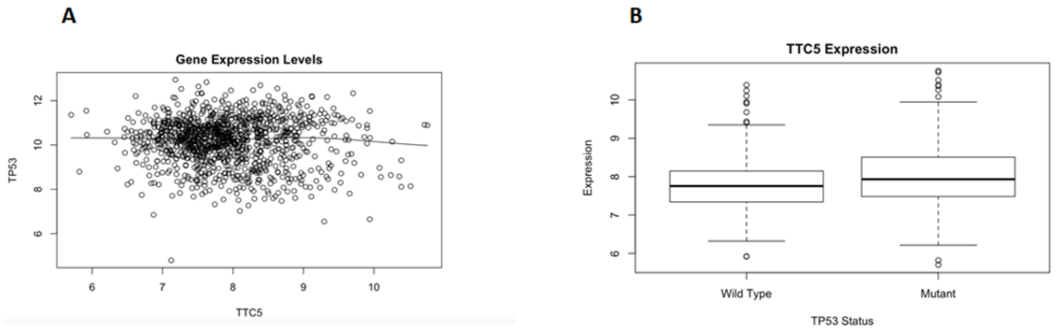

Supplementary figure S3 P53 mRNA expression vs TTC5 mRNA expression from TCGA. Expression data for TTC5 and TP53 from the TCGA LUNG database were used to compare the expression levels of the wild type TP53 and TTC5 mRNA and displayed in a scatter graph (A). TTC5 expression was compared to mutant or wild-type TP53 as shown in a boxplot (B).

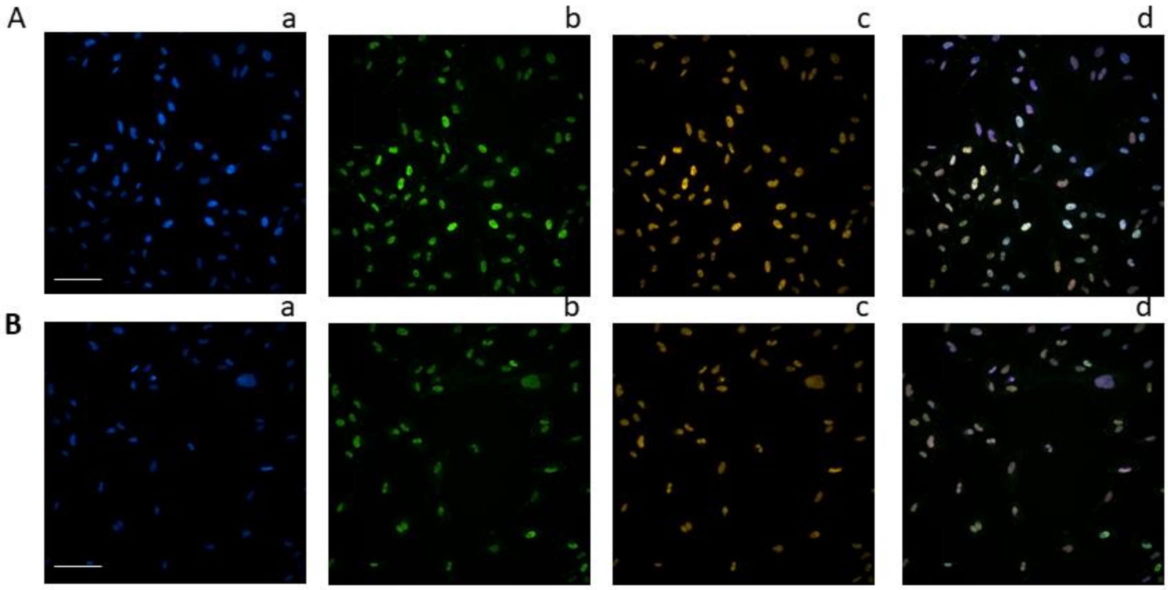

Supplementary figure S4 The location of total and p53 acetylated at lysine 382 proteins in not treated (A) and Etoposide treated (B) BEAS 2B cell lines. The BEAS 2B cells were treated with 20µM Etoposide for 24hrs and analysed using immunofluorescence. Blue DAPI staining (a). Green staining for p53 protein stained with DO-1 antibody (b). Red staining for p53 protein acetylated at K382 (c). Merged images of cells stained with DAPI, DO-1 and K382 (d). Magnification 20X was applied to all slides. Scale bars indicate 100µm.

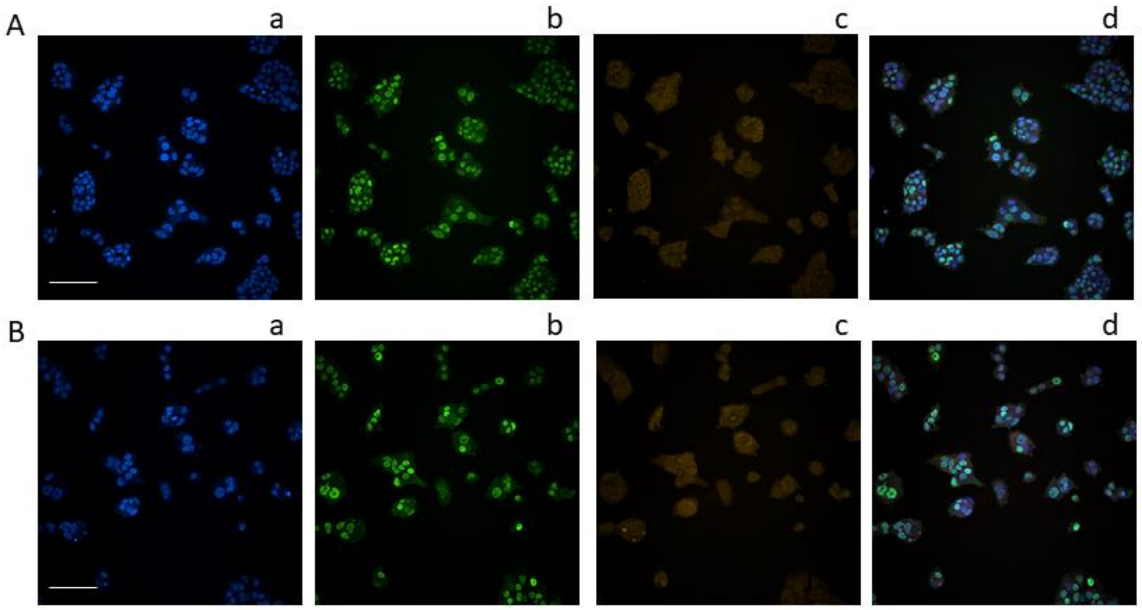

Supplementary figure S5 The location of total and p53 acetylated at lysine 382 proteins in not treated (A) and Etoposide treated (B) H2170 cell lines. The H2170 cells were treated with 20µM Etoposide for 24hrs and analysed using immunofluorescence. Blue DAPI staining (a). Green staining for p53 protein stained with DO-1 antibody (b). Red staining for p53 protein acetylated at K382 (c). Merged images of cells stained with DAPI, DO-1 and K382 (d). Magnification 20X was applied to all slides. Scale bars indicate 100µm.

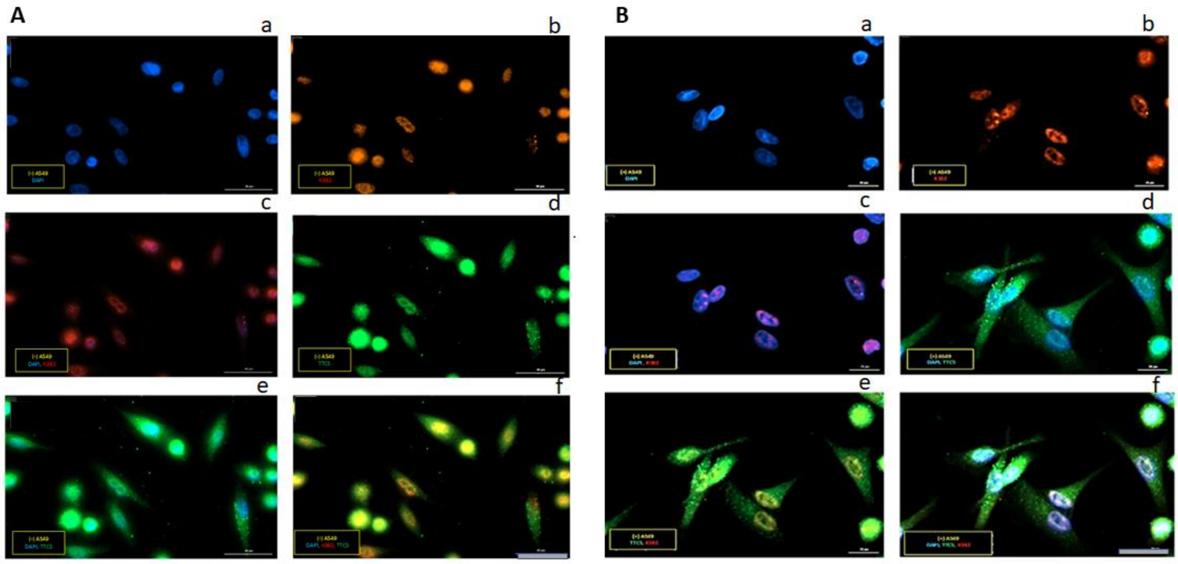

Supplementary figure S6 The location of p53 acetylated at lysine 382 and TTC5 proteins in not treated (A) and Etoposide treated (B) A549 cell lines. The A549 cells were treated with 20µM Etoposide for 24hrs and analysed using immunofluorescence. Blue DAPI staining (a). Red staining for K382 protein (b, c). Green staining for TTC5 protein (d, e). Merged images of cells stained with DAPI, K382 and TTC5 staining (f). Magnification 20X was applied to all slides. Scale bars indicate 40µm for A and 20µm for B.

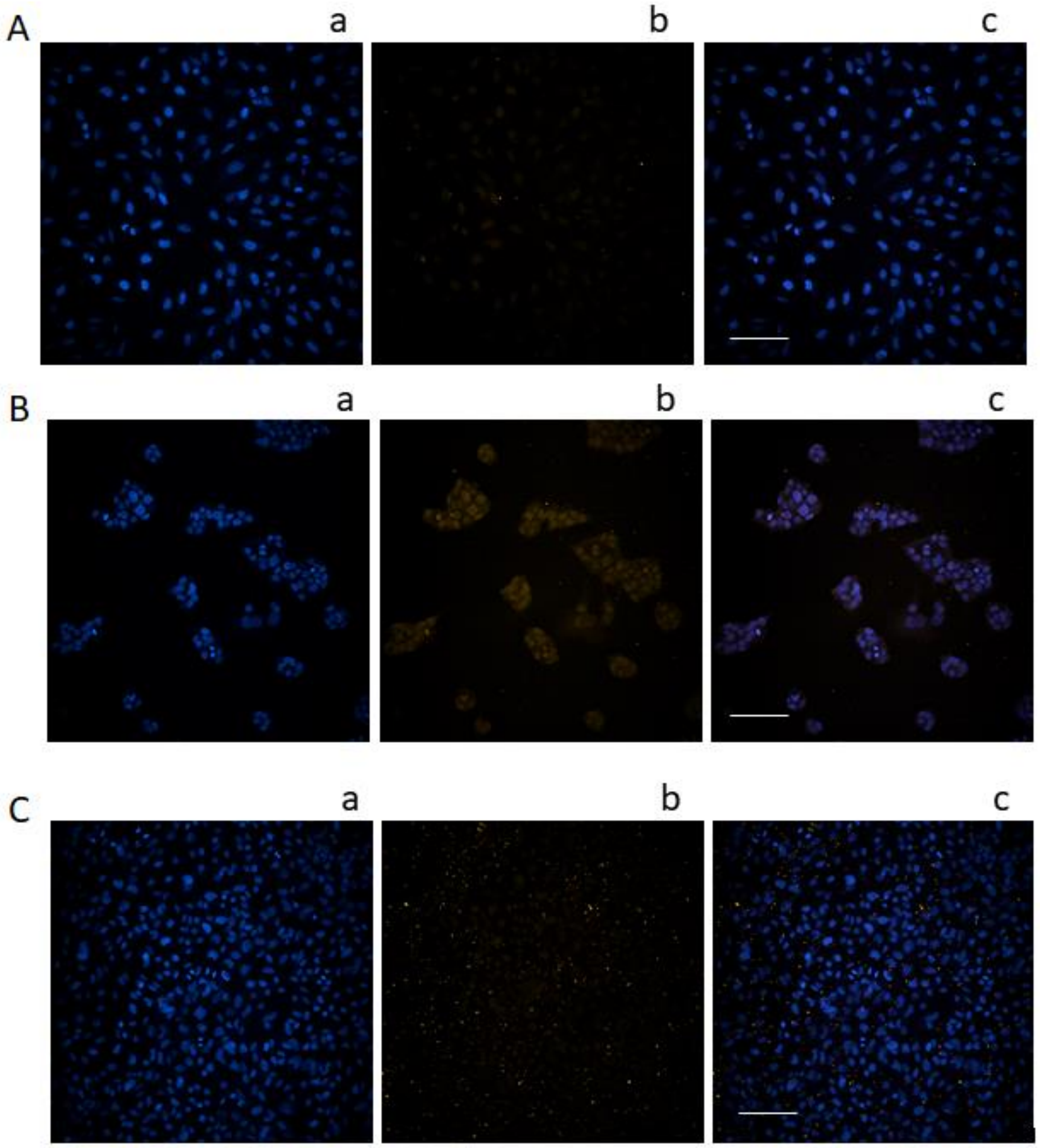

Supplementary figure S7 Lung cell lines stained with IgG control antibody. (A) BEAS 2B, (B) H2170 and A549 cell lines were stained with IgG control antibody and analysed using immunofluorescence. Blue DAPI staining (a). Red staining using IgG antibody (b). Merged images of cells stained with DAPI and IgG antibody (c). Magnification 20X was applied to all slides. Scale bars indicate 100µm.

Supplementary Table S1. Association of total p53 (Tp53) and its acetylated form (K382) with clinicopathological features

	TP53			K382		
**Characteristic**	**Nº**	**Positive**	***p*-Value**	**Nº**	**Positive**	***p*-Value**
Gender			0.332			**0.004**
Male	190 (76.0%)	127 (66.8%)		190 (76.0 %)	139 (73.2 %)	
Female	60 (24.0%)	36 (60.0%)		60 (24.0 %)	32 (53.3 %)	
Age			0.443			0.88
>60 y	147 (58.8%)	93(63.3%)		147 (58.8%)	100 (68.0 %)	
<61 y	103 (41.2%)	70 (68.0%)		103 (41.2 %)	71 (68.9 %)	
Cancer grade			**0.001**			**0.039**
Grade 1	6 (3.4%)	2 (33.3%)		6 (3.4%)	4 (66.7%)	
Grade 2	88 (50%)	58 (65.9%)		88 (50 %)	67(76.1%)	
Grade 3	51 (29.0%)	36 (70.6%)		51 (29.0%)	31 (60.8 %)	
Grade 1-2	20 (11.4%)	5 (25.0%)		20 (11.4%)	17 (85%)	
Grade 2-3	11 (6.3%)	10 (90.9%)		11 (6.3%)	11 (100%)	
Stage of the cancer			0.513			0.884
I	7 (6.4%)	4 (57.1%)		7 (6.4 %)	4 (57.1%)	
II	2 (1.8%)	1 (50%)		2 (1.8 %)	1 (50%)	
IA	4(3.6%)	3 (75.0%)		4 (3.6%)	3 (75.0 %)	
IIA	5 (4.5 %)	3 (60%)		5 (4.5 %)	3 (60.0 %)	
IIIA	27 (24.5%)	23 (85.2%)		27 (24.5%)	16 (59.3 %)	
IB	44 (4.0%)	32 (72.7%)		44 (40 %)	25 (56.8 %)	
IIB	17(15.5%)	9 (52.9%)		17 (15.5%)	7 (41.2 %)	
IIIB	3 (2.7%)	2 (66.7%)		3 (2.7 %)	1 (33.3 %)	
IV	1 (0.9 %)	1 (100%)		1 (0.9 %)	1 (100 %)	
TNM Stage						
T1	31 (20.3%)	23 (74.2%)	0.559	31 (20.3 %)	17 (54.8 %)	0.969
T2	92 (60.1%)	62 (67.4%)		92 (60.1 %)	53 (57.6 %)	
T3	23 (15.0%)	18 (78.3%)		23 (15 %)	12 (52.2 %)	
T4	7 (4.6%)	6 (85.7%)		7 (4.6 %)	4 (57.1 %)	
M0	132 (93.0%)	94 (71.2%)	0.576	132 (93.0 %)	75 (56.8%)	0.434
M1	2 (1.4%)	2 (100 %)		2 (1.4 %)	2 (100 %)	
MX	8 (5.6 %)	5 (62.5 %)		8 (5.6 %)	4 (50 %)	
N0	88 (59.5%)	64 (72.7%)	0.685	88 (59.5 %)	51 (58.0 %)	0.111
N1	55 (37.2%)	38 (69.1%)		55 (37.2 %)	30 (54.5 %)	
N2	4 (2.7%)	2 (50 %)		4 (2.7 %)	0 (0.0 %)	
NX	1 (0.7 %)	1 (100 %)		1 (0.7 %)	1 (100 %)	
P53 Intensity						**0.044**
Negative	87 (34.8 %)			87 (34.8 %)	52 (59.8%)	
Weak	74 (29.6 %)			74 (29.6 %)	50 (67.6 %)	
Moderate	41 (16.4 %)			41 (16.4 %)	29 (70.7 %)	
Strong	48 (19.2 %)			48 (19.2 %)	40 (83.3 %)	
K382 Intensity			0.202			
Negative	79 (31.6%)	44 (55.7%)		79 (31.6 %)		
Weak	92 (36.8%)	64 (69.6%)		92 (36.8 %)		
Moderate	55 (22.0%)	38 (69.1%)		55 (22.0 %)		
Strong	24 (9.6%)	17 (70.8%)		24 (9.6%)		
S46 Intensity			0.43			0.141
Negative	31 (12.4%)	17(54.8%)		31 (12.4 %)	17 (54.8%)	
Weak	99 (39.6%)	65(65.7%)		99 (39.6 %)	66 (66.7 %)	
Moderate	85 (34.0%)	55(64.7%)		85 (34.0 %)	65 (76.5%)	
Strong	35 (14.0%)	26 (74.3%)		35 (14.0 %)	23 (65.7 %)	
TTC5 Intensity			0.268			0.31
Negative	67 (26.8 %)	41 (61.2 %)		67 (26.8 %)	46 (68.7%)	
Weak	113 (45.2 %)	81 (71.7 %)		113 (45.2 %)	82 (72.6 %)	
Moderate	44 (17.6%)	26 (59.1 %)		44 (17.6 %)	29 (65.9 %)	
Strong	26 (10.4%)	15 (57.7%)		26 (10.4 %)	14 (53.8%)	
SIRT1 Intensity			0.13			0.66
Negative	15 (31.3%)	8 (53.3%)		15 (31.3 %)	8 (53.3 %)	
Weak	3 (6.3%)	1 (33.3 %)		3 (6.3 %)	1 (33.3 %)	
Moderate	9 (18.8 %)	8 (88.9 %)		9 (18.8 %)	7 (77.8 %)	
Strong	21 (43.8 %)	16 (76.2 %)		21 (43.8%)	12 (57.1 %)	
K382 Localization			0.059			
Negative	78 (31.2 %)	43 (55.1 %)		78 (31.2 %)		
Nuclear staining	108 (43.2 %)	73 (67.6 %)		108 (43.2 %)		
Cytoplasmic staining	64 (25.6 %)	47 (73.4 %)		64 (25.6 %)		

Supplementary Table S2. Association of the acetylated p53 (K382) subcellular localization with clinicopathological features

Characteristic	Total	Negative	Nuclear Staining	Cytoplasmic Staining	*p*-Value
Gender					**0.007**
Male	190 (76.0 %)	50 (26.3 %)	85 (44.7 %)	55 (28.9 %)	
Female	60 (24 %)	28 (46.7 %)	23 (38.3 %)	9 (15.0 %)	
Age					0.99
>60 y	147 (58.8 %)	46 (31.3 %)	63 (42.9 %)	38 (25.9 %)	
<61 y	103 (41.2 %)	32 (31.1 %)	45 (43.7 %)	26 (25.2 %)	
Cancer grade					**0.024**
Grade 1	6 (3.4 %)	2 (33.3 %)	2 (33.3 %)	2 (33.3 %)	
Grade 2	88 (50.0 %)	21 (23.9 %)	41 (46.6 %)	26 (29.5 %)	
Grade 3	51 (29.0 %)	20 (39.2 %)	21 (41.2 %)	10 (19.6 %)	
Grade 1-2	20 (11.4 %)	2 (10.0 %)	10 (50 %)	8 (40.0 %)	
Grade 2-3	11 (6.3 %)	0 (0.0 %)	10 (90.9 %)	1 (9.1 %)	
Stage of the cancer					0.984
I	7 (6.4 %)	3 (42.9 %)	3 (42.9 %)	1 (14.3 %)	
II	2 (1.8 %)	1 (50 %)	1 (50 %)	0 (0.0%)	
IA	4 (3.6 %)	1 (25 %)	3 (75 %)	0 (0.0 %)	
IIA	5 (4.5 %)	2 (40 %)	2 (40 %)	1 (20.0 %)	
IIIA	27 (24.5 %)	11 (40.7 %)	12 (44.4 %)	4 (14.8 %)	
IB	44 (40 %)	19 (43.2%)	20 (45.5 %)	5 (11.4 %)	
IIB	17 (15.5 %)	10 (58.8%)	6 (35.3 %)	1 (5.9 %)	
IIIB	3 (2.7 %)	2 (66.7 %)	1 (33.3 %)	0 (0.0%)	
IV	1 (0.9 %)	0 (0.0 %)	1 (100 %)	0 (0.0%)	
TNM Stage					
T1	31 (20.3 %)	14 (45.2 %)	10 (32.3%)	7 (22.6 %)	0.91
T2	92 (60.1 %)	39 (42.4 %)	40 (43.5%)	13 (14.1 %)	
T3	23 (15.0 %)	11 (47.8 %)	8 (34.8 %)	4 (17.4 %)	
T4	7 (4.6 %)	3 (42.9 %)	3 (42.9 %)	1 (14.3 %)	
M0	132 (93.0 %)	57 (43.2 %)	53 (40.2 %)	22 (16.7 %)	0.539
M1	2 (1.4 %)	0 (0.0 %)	2 (100 %)	0 (0.0 %)	
MX	8 (5.6 %)	4 (50 %)	3 (37.5 %)	1 (12.5 %)	
N0	88 (59.5 %)	37 (42.0 %)	34 (38.6 %)	17 (19.3 %)	0.261
N1	55 (37.2 %)	25 (45.5 %)	23 (41.8 %)	7 (12.7 %)	
N2	4 (2.7 %)	4 (100%)	0 (0.0 %)	0 (0.0 %)	
NX	1 (0.7 %)	0 (0.0 %)	1 (100 %)	0 (0.0 %)	

Supplementary Table S3. Association of the phosphorylated p53 (Ser-46) with clinicopathological features

	S46		
**Characteristic**	**N°**	**Positive**	***p*-Value**
Gender			0.25
Male	190 (76.0 %)	169 (88.9 %)	
Female	60 (24.0 %)	50 (83.3 %)	
Age			0.385
>60 y	147 (58.8 %)	131 (89.1 %)	
<61 y	103 (41.2 %)	88 (85.4 %)	
Cancer grade			**0.019**
Grade 1	6 (3.4 %)	6 (100 %)	
Grade 2	88 (50.0 %)	84 (95.5%)	
Grade 3	51 (29.0 %)	42 (82.4%)	
Grade 1-2	20 (11.4%)	20 (100%)	
Grade 2-3	11 (6.3 %)	11 (100%)	
Stage of the cancer			0.643
I	7 (6.4 %)	5 (71.4%)	
II	2 (1.8%)	1 (50.0%)	
IA	4 (3.6%)	4 (100 %)	
IIA	5 (4.5 %)	3 (60.0%)	
IIIA	27 (24.5 %)	22 (81.5%)	
IB	44 (40.0%)	38 (86.4%)	
IIB	17 (15.5%)	14 (82.4%)	
IIIB	3 (2.7 %)	3 (100%)	
IV	1 (0.9 %)	1 (100%)	
TNM Stage			
T1	31 (20.3%)	24 (77.4 %)	0.876
T2	92 (60.1%)	77 (83.7%)	
T3	23 (15.0%)	19 (82.6%)	
T4	7 (4.6 %)	6 (85.7 %)	
M0	132 (93.0 %)	108 (81.8 %)	0.74
M1	2 (1.4 %)	2 (100 %)	
MX	8 (5.6 %)	7 (87.5 %)	
N0	88 (59.5 %)	74 (84.1 %)	0.474
N1	55 (37.2 %)	42 (76.4 %)	
N2	4 (2.7 %)	4 (100 %)	
NX	1 (0.7 %)	1 (100 %)	
P53 Intensity			0.413
Negative	87 (34.8 %)	73 (83.9%)	
Weak	74 (29.6 %)	67 (90.5 %)	
Moderate	41 (16.4 %)	38 (92.7 %)	
Strong	48 (19.2 %)	41 (85.4 %)	
K382 Intensity			0.177
Negative	79 (31.6 %)	65 (82.3 %)	
Weak	92 (36.8 %)	80 (87.0 %)	
Moderate	55 (22.0 %)	51 (92.7 %)	
Strong	24 (9.6 %)	23 (95.8 %)	
S46 Intensity			
Negative	31 (12.4 %)		
Weak	99 (39.6 %)		
Moderate	85 (34.0 %)		
Strong	35 (14.0 %)		
TTC5 Intensity			0.868
Negative	67 (26.8%)	58 (86.6%)	
Weak	113 (45.2%)	99 (87.6%)	
Moderate	44 (17.6%)	40 (90.9%)	
Strong	26 (10.4%)	22 (84.6 %)	
SIRT1 Intensity			0.312
Negative	15 (31.3 %)	11 (73.3 %)	
Weak	3 (6.3 %)	2 (66.7%)	
Moderate	9 (18.8%)	9 (100 %)	
Strong	21 (43.8 %)	18 (85.7 %)	
K382 Localization			0.198
Negative	78 (31.2 %)	64 (82.1 %)	
Nuclear staining	108 (43.2 %)	97 (89.8 %)	
Cytoplasmic staining	64 (25.6 %)	58 (90.6 %)	

Supplementary Table S4. Association of SIRT1 and TTC5 protein levels with clinicopathological features

	SIRT1			TTC5		
**Characteristic**	**N°**	**Positive**	***p*-Value**	**N°**	**Positive**	***p*-Value**
Gender			0.968			0.329
Male	19 (39.6%)	13 (68.4 %)		190 (76.0 %)	142 (74.7 %)	
Female	29 (60.4 %)	20 (69.0 %)		60 (24.0 %)	41 (68.3 %)	
Age			0.881			0.055
>60 y	39 (81.3 %)	27 (69.2 %)		147 (58.8%)	101 (68.7 %)	
<61 y	9 (18.8 %)	6 (66.7 %)		103 (41.2%)	82 (79.6 %)	
Cancer grade	NA	NA	NA			**0.039**
Grade 1	NA	NA	NA	6 (3.4 %)	5 (83.3 %)	
Grade 2	NA	NA	NA	88 (50.0 %)	73 (83.0 %)	
Grade 3	NA	NA	NA	51 (29.0 %)	43 (84.3 %)	
Grade 1-2	NA	NA	NA	20 (11.4 %)	12 (60.0 %)	
Grade 2-3	NA	NA	NA	11 (6.3 %)	6 (54.5 %)	
Stage of the cancer	NA	NA	NA			0.889
I	NA	NA	NA	7 (6.4 %)	6 (85.7 %)	
II	NA	NA	NA	2 (1.8 %)	2 (100 %)	
IA	NA	NA	NA	4 (3.6 %)	4 (100 %)	
IIA	NA	NA	NA	5 (4.5 %)	5 (100 %)	
IIIA	NA	NA	NA	27 (24.5 %)	26 (96.0 %)	
IB	NA	NA	NA	44 (40.0%)	41 (93.2 %)	
IIB	NA	NA	NA	17 (15.5 %)	17 (100 %)	
IIIB	NA	NA	NA	3 (2.7 %)	3 (100 %)	
IV	NA	NA	NA	1 (0.9 %)	1 (100 %)	
TNM Stage						
T1	22 (51.2 %)	17 (77.3 %)	0.229	31 (20.3%)	17 (54.8 %)	**<0.001**
T2	14 (32.6 %)	9 (64.3 %)		92 (60.1 %)	81 (88.0 %)	
T3	4 (9.3 %)	1 (25.0 %)		23 (15.0 %)	21 (91.3 %)	
T4	3 (7 %)	2 (66.7 %)		7 (4.6 %)	5 (71.4 %)	
M0	23 (71.9 %)	12 (52.2 %)	0.151	132 (93.0 %)	115 (87.1 %)	**<0.001**
M1	1 (3.1 %)	1 (100%)		2 (1.4 %)	1 (50.0 %)	
MX	8 (25.0 %)	7 (87.5 %)		8 (5.6 %)	2 (25.0 %)	
N0	30 (78.9%)	22 (73.3 %)	0.082	88 (59.5 %)	64 (72.7 %)	**0.013**
N1	3 (7.9 %)	1 (33.3 %)		55 (37.2 %)	52 (94.5 %)	
N2	4 (10.3 %)	1 (25.0 %)		4 (2.7 %)	3 (75 %)	
NX	1 (2.6 %)	0 (0.0 %)		1 (0.7 %)	1 (100%)	
P53 Intensity			0.453			0.484
Negative	15 (31.3 %)	8 (53.3 %)		87 (34.8 %)	61 (70.1 %)	
Weak	19 (39.6 %)	15 (78.9 %)		74 (29.6 %)	52 (70.3 %)	
Moderate	7 (14.6 %)	5 (71.4 %)		41 (16.4 %)	31 (75.6 %)	
Strong	7 (14.6 %)	5 (71.4 %)		48 (19.2 %)	39 (81.3%)	
K382 Intensity			0.878			0.979
Negative	20 (41.7 %)	13 (65.0 %)		79 (31.6 %)	58 (73.4 %)	
Weak	25 (52.1 %)	18 (37.5 %)		92 (36.8 %)	66 (71.7%)	
Moderate	3 (6.3 %)	2 (66.7 %)		55 (22.0 %)	41 (74.5 %)	
Strong	/	/		24 (9.6 %)	18 (75.0%)	
S46 Intensity			**0.004**			0.379
Negative	8 (16.7 %)	4 (50.0 %)		31 (12.4 %)	22 (71.0 %)	
Weak	11 (22.9 %)	4 (63.4 %)		99 (39.6 %)	69 (69.7 %)	
Moderate	15 (31.3 %)	11 (73.3 %)		85 (34.0 %)	68 (80 %)	
Strong	14 (29.2 %)	14 (100 %)		35 (14.0 %)	24 (68.6 %)	
TTC5 Intensity			**0.019**			
Negative	27 (56.3 %)	23 (85.2 %)				
Weak	16 (33.3 %)	8 (50.0 %)				
Moderate	5 (10.4 %)	2 (40.0 %)				
Strong	/	/				
SIRT1 Intensity						**0.044**
Negative	15 (31.3 %)			15 (31.3 %)	11 (73.3 %)	
Weak	3 (6.3 %)			3 (6.3 %)	1 (33.3 %)	
Moderate	9 (18.8 %)			9 (18.8 %)	2 (22.2 %)	
Strong	21 (43.8 %)			21 (43.8 %)	7 (33.3 %)	
K382 Localization			0.87			0.645
Negative	20 (41.7 %)	16 (65.0 %)		108 43.2 %)	81 (75.0 %)	
Nuclear staining	13 (27.1 %)	9 (69.2 %)		64 (25.6 %)	44 (68.8 %)	
Cytoplasmic staining	15 (31.3 %)	11 (73.3 %)		78 (31.2 %)	58 (74.4 %)	

Supplementary Table S5 Summary of analysis and clinical data (attached separately)
